# Botanical characterization, phytochemistry, biosynthesis, pharmacology clinical application, and breeding techniques of the Chinese herbal medicine *Fritillaria unibracteata*

**DOI:** 10.3389/fphar.2024.1428037

**Published:** 2024-07-09

**Authors:** Yamei Zhang, Hongping Han, Dingai Li, Yanan Fan, Meng Liu, Huimin Ren, Lu Liu

**Affiliations:** ^1^ School of Chemistry and Chemical Engineering, Qinghai Normal University, Xining, China; ^2^ Key Laboratory of Medicinal Plant and Animal Resources of Qinghai-Tibetan Plateau in Qinghai Province, Xining, China; ^3^ Academy of Plateau Science and Sustainability, People’s Government of Qinghai Province, Xining, China

**Keywords:** *Fritillaria unibracteata*, botanical characterization, phytochemistry, biosynthesis, pharmacological effects, clinical application, breeding techniques

## Abstract

*Fritillaria unibracteata* (FRU) belongs to the genus *Fritillaria* of the Liliaceae family. It is one of the original plants of the Chinese medicinal material “Chuanbeimu” and also a biological resource featured in the Tibetan Plateau of China. The dried bulbs of FRU are used in traditional Chinese medicine. The chemical constituents of FRU that have been isolated and identified include alkaloids, sterols, organic acids and their esters, nucleosides and volatile oils. FRU has antitussive, expectorant, anti-asthmatic, anti-inflammatory, antibacterial, acute lung injury-reducing, antifibrosis, antitumor, and other pharmacological effects. This valuable plant has an extremely high market demand, and over the years, due to over-exploitation, FRU has now been listed as a key species that is endangered and scarcely cultivated in China as a traditional Chinese medicinal herb. However, research on FRU is rare, and its effective components, resource control, and mechanisms of action need further study. This review systematically discusses the herbal characteristics, resource distribution, chemical composition, biosynthesis, pharmacological effects, clinical application, and breeding techniques of FRU, hoping to provide a reference for further research and the use of FRU.

## 1 Introduction

Chuanbeimu (CBM) consists of the desiccated bulbs of *Fritillaria cirrhosa* D. Don (FCD), *F. unibracteata* Hsiao et K.C. Hsia (FRU), *F. przewalskii* Maxim. (FRP), *F. delavayi* Franch. (FRD), *F. taipaiensis* P.Y.Li (FRT) or *F. unibracteata* Hsiao et K.C. Hsiavar wabuensis (FRW) (Chinese Pharmacopoeia Commission, 2020). As one of the main plants included in CBM, FRU is a characteristic biological resource of the Tibetan Plateau, and is known as the “Holy Medicine for cough,” with the effects of clearing heat and moistening the lungs, resolving phlegm, relieving cough and resolving masses, and it is widely used in traditional Chinese medicine (TCM) for the treatment of diseases of the respiratory system (Guo et al., 2016). The most popular product, Chuanbei Pipa syrup, has an annual sales volume of more than US$70 million and has gained international recognition and is sold in more than 20 countries. One of the raw materials used in Chuanbei Pipa syrup is FRU (Cunning et al., 2018; (Gao et al., 2018). FRU is one of the medicinal materials in the traditional Chinese medicine prevention and treatment plan of COVID-19 issued by the Qinghai Province in 2021. Modern pharmacological studies have also shown that the dried bulbs of FRU have anti-inflammatory (Wang et al., 2016), anti-asthmatic (Li et al., 2017), antibacterial (Chen et al., 2017), anti-fibrotic (Yuan et al., 2022), and anti-tumor effects (Yun et al., 2008), as well as being effective for reducing acute lung injury (Guo et al., 2013). With the development of modern techniques for isolation and structural analysis, FRU has been found to contain a variety of components, including alkaloids (Zhang et al., 2011), sterols (Yu and Xiao, 1990a), organic acids and their esters (Yun et al., 2009), nucleosides (Zhang et al., 2012), and volatile oils (Guo et al., 2022). Of these, alkaloids form the major active components and have been the most studied. However, due to the extremely high medicinal value of FRU, its market demand continues to expand, wild FRU resources have been overexploited, while the price has increased from RMB 100/kg in 2004 to a current high of RMB 5000/kg. It has now been listed as a national third-grade endangered medicinal material for protection. Thus, the cultivation of FRU that has efficacy comparable with that of wild resources is a major difficulty that currently needs to be solved. In recent years, in order to better utilize FRU as a rare resource, effectively promote the study of its pharmacological effects, and explore its clinical applications, there has been significant investment in the research into FRU. However, despite progress in the field, there has been no comprehensive and systematic overview of current research findings. Therefore, here, we provide a systematic review of the botanical characterization, resource distribution, phytochemistry, biosynthesis, pharmacological activity, clinical application, and breeding techniques of FRU to provide a reference for the development and utilization of FRU-containing resources.

## 2 Botanical characterization and distribution of resources

As a precious traditional Chinese medicine, the earliest understanding of the ancient texts of Bei Mu can be traced back to the Spring and Autumn Period, and the name “Meng” has been recorded in the *Book of Poetry*. It was first recorded in *Shennong’s Classic of Materia Medica* by the name Bei Mu and is listed as a middle-grade herbal medicine that can clear heat and relieve cough and inflammation (Zhao et al., 2020). The origin of the name Bei Mu has been explained in *The Collection of Bencao Jing* of Tao Hongjing in the Liang Dynasty: “The shape is like a collection of shellfish, so it is called Bei Mu (Wu et al., 2022).” Both *Zheng Lei Ben Cao* in the Northern Song Dynasty and *Ben Cao Shu* in the Qing Dynasty described the growth period, stems, and flower morphology of Bei Mu as follows: “the color is yellowish white, shape is like a collection of shellfish, so it is called Bei Mu (An et al., 2022).” Since the Tang Dynasty, Bei Mu has been used as traditional Chinese medicine to this day. FRC first appeared in *Bencao Huiyan*, where it was used to further categorize and compare the types of Bei Mu (Chen et al., 2020). In the Qing Dynasty, Zhao Xuemin’s *Compendium of Materia Medica* referred to the lotus flower, which is “Songbei” in today’s FRC, especially the two petals of the pine shell. The size of the scaly leaves is very different; the large petals wrap around the small petals, forming the shape of “holding the Moon in the arms (An et al., 2022).”

**FIGURE 1:**
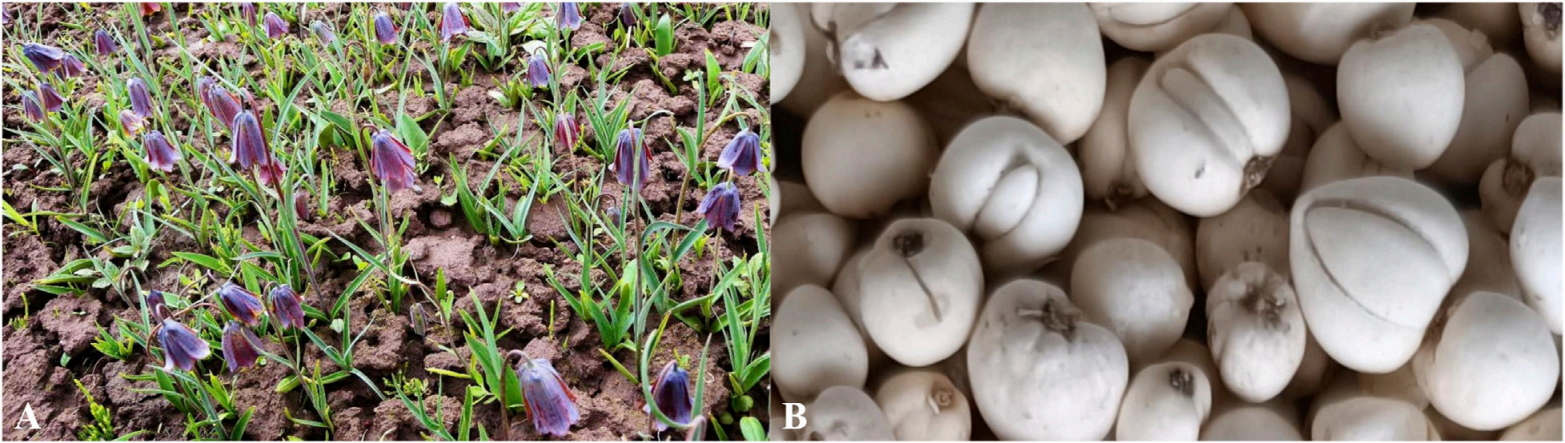
**(A)** Flowers and **(B)** bulbs of FRU.

As a perennial herb, the main source of Songbei, FRU grows in alpine meadows and shrubs at an altitude of 3,200–4,500 m, mainly in the Zoige Plateau and the eastern section of the western Sichuan mountains and valleys, including the counties of Aba Prefecture in the Sichuan Province and Guoluo and Banma in the Qinghai Province (Chen et al., 1997; Guo et al., 2016). It grows well in cold climates where there is strong sunshine, e.g., in spring and autumn. The diameter of the FRU bulb is approximately 1 cm, and the smaller bulb is preferred. The main difference in morphology from other types of FRU is that its flowers are dark purple and the tepals have small yellowish-brown squares (Editorial Committee of Flora of ChinaChinese Academy of Sciences, 2004). The flowers and bulbs of FRU are shown in Figure 1. The growth rate of FRU bulbs is particularly slow, and obtaining commercial medicinal materials from seeds generally takes 4 years (Yun and Chen, 2010).

## 3 Phytochemistry

### 3.1 Chemical composition

**FIGURE 2:**
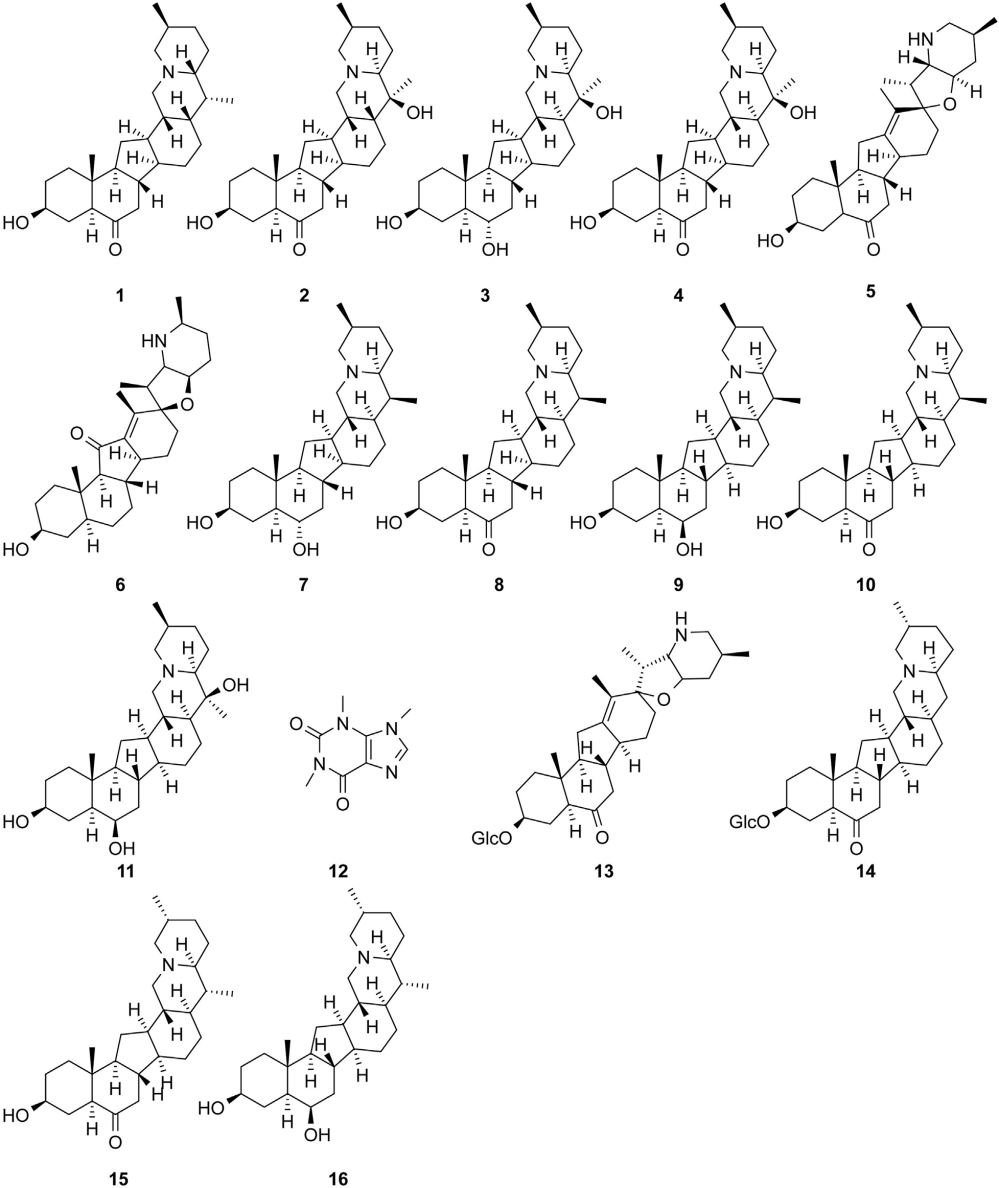
Chemical structure of alkaloid (compounds 1–16) in FRU.

**FIGURE 3:**
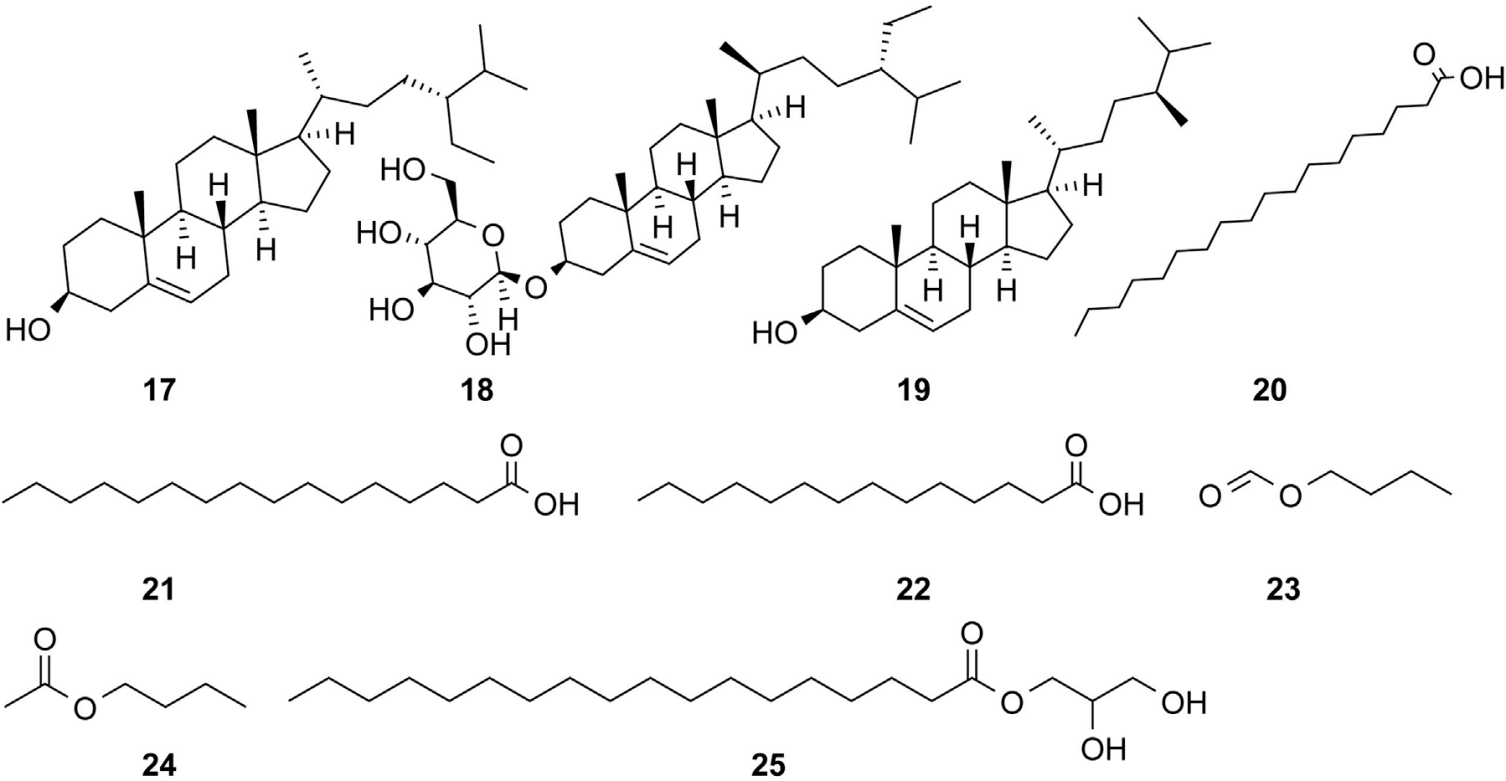
Chemical structure of sterols and organic acids (compounds 17–25) in FRU.

**FIGURE 4:**
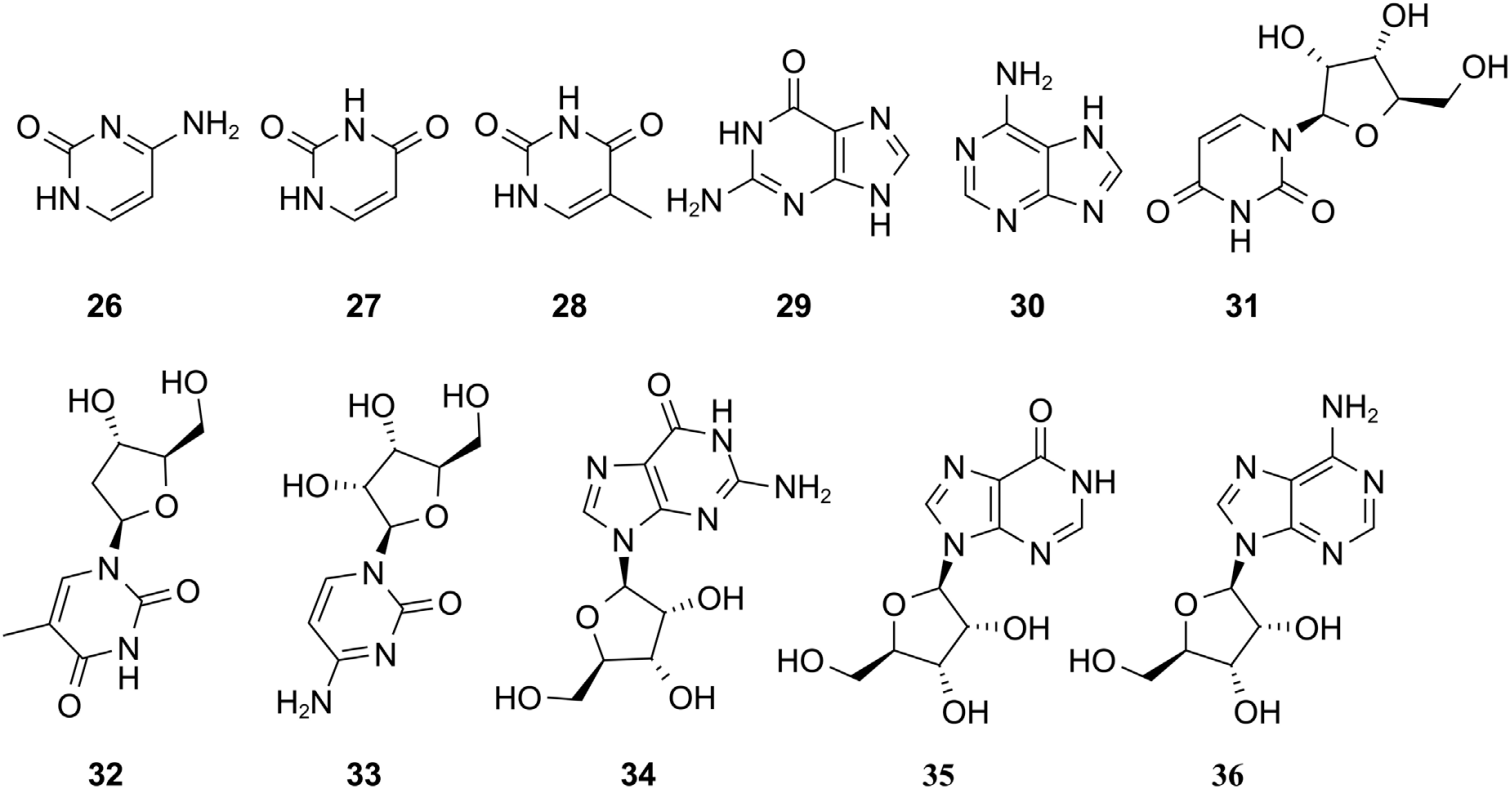
Chemical structure of nucleosides (compounds 26–36) in FRU.

**FIGURE 5:**
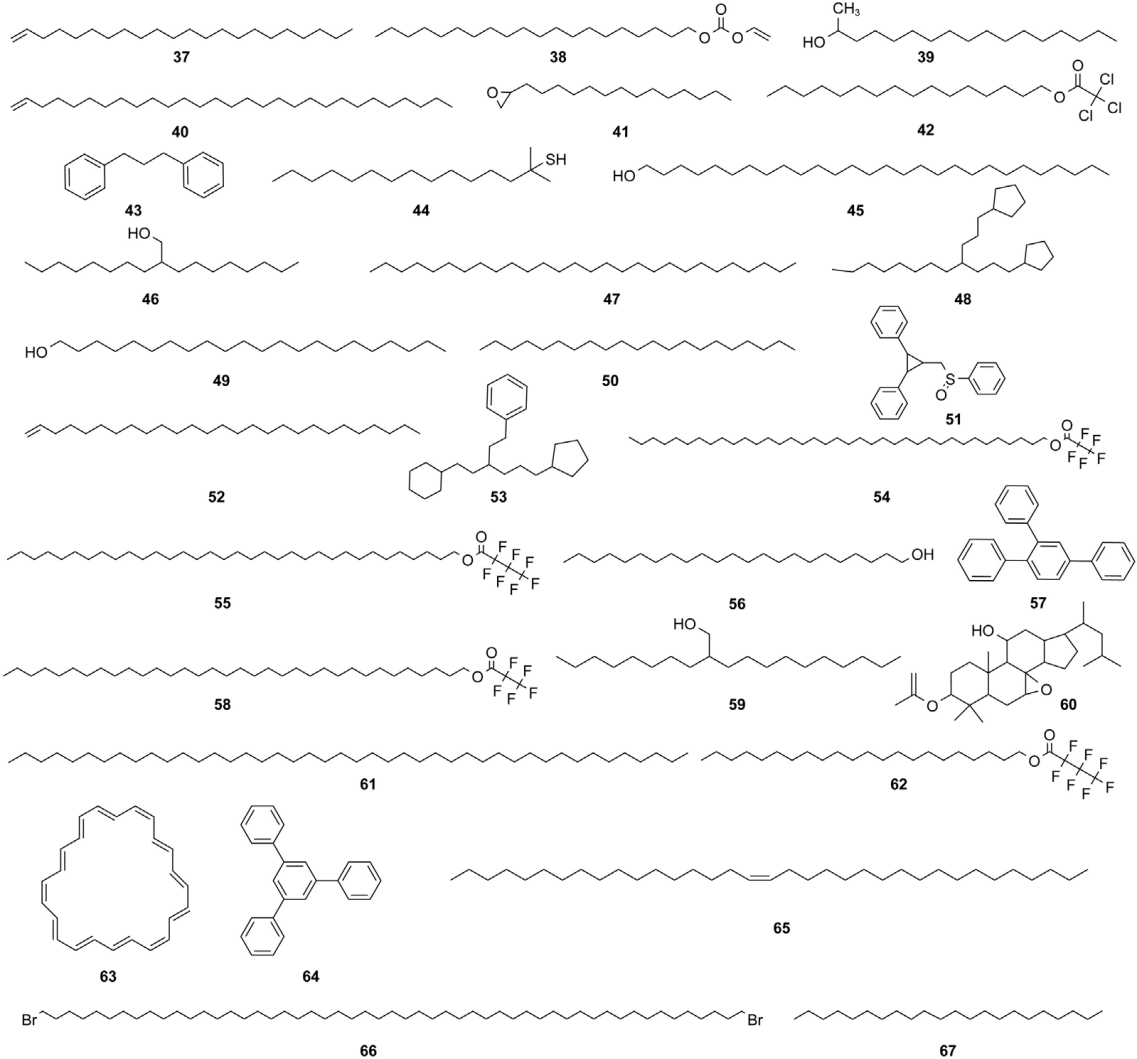
Chemical structure of volatile oils (compounds 37–67) in FRU.

**FIGURE 6:**
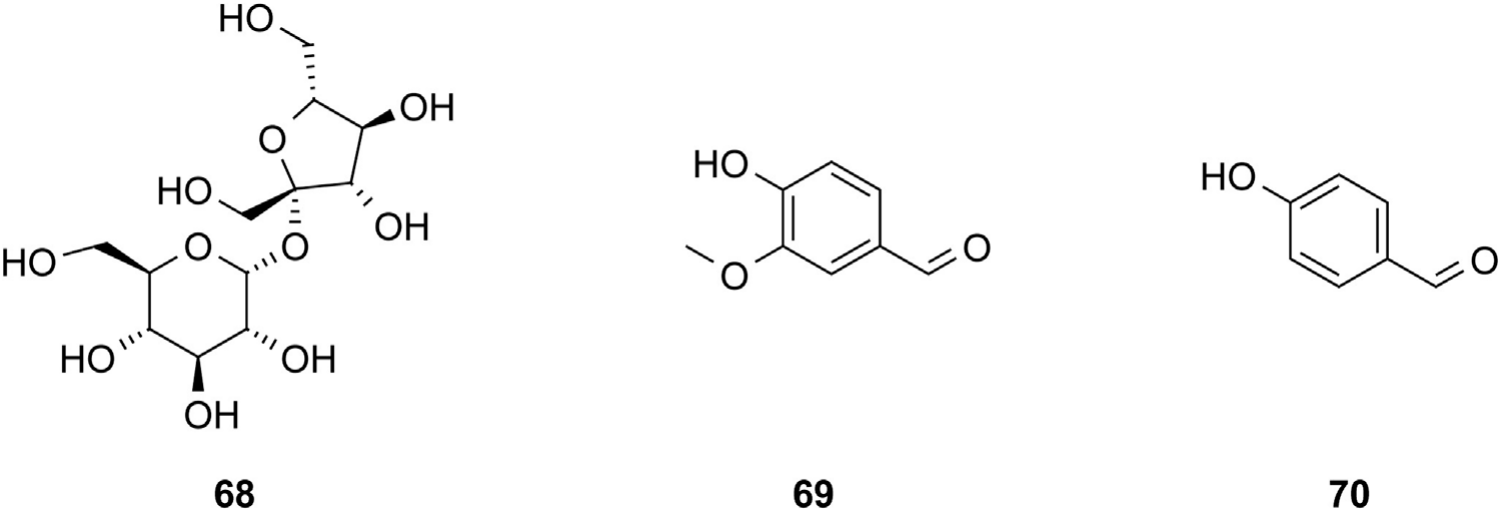
Chemical structure of other compounds (compounds 68–70) in FRU.

The study of the chemical constituents of *Fritillaria* began in 1888 when Fragner isolated imperialine from *F. imperialis* L. Subsequently, an increasing number of German, Japanese, and Chinese chemists began to participate in this study (Yun and Chen, 2010). After several years of research on *Fritillaria*, Zhu et al. made great progress in communicating the relationship between *Fritillaria* alkaloids and veratrine alkaloids. In 1955, using the method of selenium dehydrogenation and degradation, they determined that the basic skeleton of alkaloids in *Fritillaria* was metamorphic steroids (Lai, 2014). *Fritillaria* contains several chemical components that can be divided into alkaloids and nonalkaloids. Alkaloids are an important component responsible for its pharmacological activity and are also the most studied substance (Qi et al., 2023). The study on the chemical constituents of FRU was first reported in 1986 wherein Kaneko et al. (1986) extracted chuanbeinone from FRU. From 1990 to 1992, Yu et al. isolated songbeisine, songbeinine, and songbeinone from FRU, which promoted the study of the chemical constituents of FRU (Yu and Xiao, 1990a; Yu and Xiao, 1990b; Yu et al., 1992). In 1999, Li et al. (1999) identified ebeidine, ebeiedinone, verticine, isoverticine, verticinone, and imperialine in FRU. In 2016, Zhou et al. (2016) isolated caffeine from FRU by repeated silica gel column chromatography. According to the literature, the nonalkaloid components of FRU include β-sitosterol, D-sucrose, butyl formate, palmitic acid, n-docosane, carbonic acid eicosyl vinyl ester, vanillin, p-hydroxybenzaldehyde, and glycerol monostearate among other compounds. Moreover, K, Na, Ca, Mg, Fe, Mn, Cr, Ni, Pb, Cd, and other trace elements are also present (Yu and Xiao, 1990a; Yun et al., 2008; Yun and Chen, 2010; Zhao et al., 2012; Guo et al., 2022). The main compounds in FRU are listed in Table 1. The alkaloids (compounds 1–16) isolated and identified from FRU are shown in Figure 2, sterols and organic acids (compounds 17–25) are shown in Figure 3, nucleosides (compounds 26–36) are shown in Figure 4, volatile oils (compounds 37–67) are shown in Figure 5, and other compounds (compounds 68–70) as shown in Figure 6.

**TABLE 1:** Main compounds in FRU.

Category	No.	Compound name	References
Alkaloid compounds	1	Chuanbeinone	Kaneko et al. (1986)
	2	Imperialine	Li et al. (1999)
	3	Peimine/Verticine	Li et al. (1999); Zhao et al., 2012
	4	Peiminine/Verticinone	Li et al. (1999); Zhao et al. (2012)
	5	Peimisine	Zhang et al. (2011)
	6	Songbeisine	Yu and Xiao. (1990a)
	7	Songbeinine	Yu and Xiao. (1990b)
	8	Songbeinone	Yu et al. (1992)
	9	Ebeiedine	Li et al. (1999)
	10	Ebeiedinone	Li et al. (1999)
	11	Isoverticine	Li et al. (1999)
	12	Caffeine	Zhou et al. (2016)
	13	Peimisine-3-O-β-D-glucopyranoside	Zhang et al. (2011)
	14	Puqiedinone-3-O-β-D-glucopyranoside	Zhang et al. (2011)
	15	Puqiedinone	Zhang et al. (2011)
	16	Puqiedine	Zhang et al. (2011)
Sterols compounds	17	β-sitosterol	Yu and Xiao. (1990a)
	18	Daucosterol	Zhao et al. (2012)
	19	Campesterol	Yun and Chen. (2009)
Organic acids and their ester compounds	20	Stearic acid	Yu and Xiao, 1990a; Yun and Chen, 2009
	21	Palmitic acid	Yu and Xiao, 1990b; Yun and Chen, 2009
	22	Myristic acid	Yun and Chen. (2009)
	23	Butyl formate	Yun and Chen. (2009)
	24	Butyl acetate	Yun and Chen. (2009)
	25	Glycerol monostearate	Zhou et al. (2016)
Nucleosides compounds	26	Cytosine	Zhou et al. (2016)
	27	Uracil	Zhang et al. (2012)
	28	Thymine	Zhang et al. (2012)
	29	Guanine	Zhang et al. (2012)
	30	Adenine	Zhang et al. (2012)
	31	Uridine	Zhang et al. (2012)
	32	Thymidine	Zhang et al. (2012)
	33	Cytidine	Zhang et al. (2012)
	34	Guanosine	Zhang et al. (2012)
	35	Inosine	Zhang et al. (2012)
	36	Adenosine	Zhang et al. (2012)
Volatile oils compounds	37	1-Docosene	Guo et al. (2022)
	38	Carbonic acid eicosyl vinyl ester	Guo et al. (2022)
	39	2-Methyl-1-hexadecanol	Guo et al. (2022)
	40	Nonacos-1-ene	Guo et al. (2022)
	41	1,2-Epoxyhexadecane	Guo et al. (2022)
	42	Hexadecyl 2,2,2-trichloroacetate	Guo et al. (2022)
	43	1,3-Diphenylpropane	Guo et al. (2022)
	44	Tert-hexadecyl mercaptan	Guo et al. (2022)
	45	Octacosanol	Guo et al. (2022)
	46	2-Octyl-1-decanol	Guo et al. (2022)
	47	Octacosane	Guo et al. (2022)
	48	1-cyclopentyl-4- (3-cyclopentyl propyl) dodecane	Guo et al. (2022)
	49	Docosanol	Guo et al. (2022)
	50	Heneicosane	Guo et al. (2022)
	51	Trans-(2,3-diphenylcyclopropyl)methylphenyl sulfoxide	Guo et al. (2022)
	52	Heptacos-1-ene	Guo et al. (2022)
	53	[3-(2-Cyclohexylethyl)-6-Cyclopentylhexyl]-Benzene	Guo et al. (2022)
	54	Octatriacontyl pentafluoropropionate	Guo et al. (2022)
	55	Tetratriacontyl heptafluorobutyrate	Guo et al. (2022)
	56	1-Tricosanol	Guo et al. (2022)
	57	1,2,4-Triphenylbenzene	Guo et al. (2022)
	58	Tetratriacontyl pentafluoropropionate	Guo et al. (2022)
	59	2-Octyl-1-dodecanol	Guo et al. (2022)
	60	3-acetoxy-7,8-epoxide-11-alcoho	Guo et al. (2022)
	61	Tetratetracontane	Guo et al. (2022)
	62	Docosyl heptafluorobutyrate	Guo et al. (2022)
	63	1,3,5,7,9,11,13,15,17,19,21,23-dodecaen	Guo et al. (2022)
	64	1,3,5-Triphenylbenzene	Guo et al. (2022)
	65	17-Pentatriacontene	Guo et al. (2022)
	66	1,54-Dibromotetradecane	Guo et al. (2022)
	67	N-Docosane	Guo et al. (2022)
Other compounds	68	D-sucrose	Zhou et al. (2016)
	69	vanillin	Zhou et al. (2016)
	70	P-hydroxybenzaldehyde	Yun and Chen. (2009)

### 3.2 Chemical composition analysis

#### 3.2.1 Alkaloid composition analysis

It has long been customary to use the total alkaloid content as the standard for the quality evaluation of *Fritillaria*. Currently, the main methods for analyzing the total alkaloid content of FRU include titration and acid dye colorimetry (Yu and Xiao, 1990a). With the development of modern analytical techniques, thin layer chromatography (TLC), GC, HPLC, capillary electrophoresis, MS, LC-MS, and molecular imprinting-chemiluminescence analysis have been used for the analysis of monomer alkaloids. Wang and Chen (2016) used acid dye colorimetry with 0.05% bromocresol green solution as a dye to determine the total alkaloids in FRU and found the method to be accurate and precise. Li et al. (2001) established a method to determine the isosteroidal alkaloids in *Fritillaria* by HPLC-evaporative light scattering detector using acetonitrile-methanol-water (66.5:3.5:30, v/v) containing 0.006% triethylamine as the mobile phase and isolated 8 alkaloids. The method was simple, accurate, and had good reproducibility and sensitivity. Liu et al. (2023a) identified 9 major isosteroidal alkaloids in 41 batches of FRC bulbs and 17 batches of *F. pallidiflora* Schrenk bulbs using LC-MS/MS. They classified and distinguished these alkaloids based on hierarchical cluster analysis and principal component analysis. These methods had a good linear relationship and high precision. Han et al. established a new molecular imprinting–chemiluminescence analysis method for the determination of peimine, peiminine, and peimisine. This method had high sensitivity and good stability and could selectively separate alkaloids from *Fritillaria* (Han et al., 2016; Han et al., 2017; Han et al., 2017). Han et al. (2023) established an analytical method for the determination of peimine, peiminine, peimisine, and sipeimine in FRU using UPLC-TQD-MS/MS, by gradient elution with acetonitrile-0.05% aqueous ammonia as the mobile phase. This method could simultaneously detect all 4 alkaloidal constituents. Song et al. (2023) established a method for the quantitative analysis of peimisine and peiminine in FRU using UPLC-QTOF-MS/MS by gradient elution using acetonitrile solution with 0.1% acetic acid (containing 0.01 mol/L ammonium acetate) as the mobile phase. This method was both rapid and had high accuracy.

#### 3.2.2 Analysis of nonalkaloidal components

Nucleoside nonalkaloid contents have been mainly determined using chromatography. Cao et al. (2010) established, for the first time, an HPLC–diode array detector (DAD) method for the simultaneous quantification of 9 nucleosides and nucleobases in *Fritillaria* using gradient elution with acetonitrile-water as the mobile phase, making the analysis rapid, simple, and reliable. Zhang et al. (2012) used reverse-phase HPLC-DAD to quantitatively determine 5 nucleosides and 5 free bases in FRU using gradient elution with methanol-water as the mobile phase. This simple method has good reproducibility and precision and can be used for the quality control of FRU. The volatile oils in *Fritillaria* have potent anti-inflammatory, antibacterial, and analgesic effects. Guo et al. (2022) separated and identified the chemical constituents of FRU using GC-MS combined with NIST spectral library retrieval and obtained 39 components, thereby enhancing the findings related to the components and contents of the volatile oils of FRU.

## 4 Biosynthesis

### 4.1 Genomic and transcriptomic studies

The biosynthetic pathways and regulatory mechanisms associated with FRU alkaloids have not been clearly defined, and the paucity of reports in this area may be related to a lack of genetic information (Qi et al., 2023). To date, studies have shown that steroidal alkaloids can be synthesised in plants via both the MVA and MEP pathways (Kumar et al., 2021). Interestingly, the MEP pathway is abundant in leaf and stem tissues, while the MVA pathway is abundant in bulbs (Liao et al., 2023).

3-hydroxy-3-methylglutaryl-CoA reductase (HMGR) is considered to be the first rate-limiting enzyme in the MVA pathway. Xu et al. analysed the amino acid sequence of HMGR from FRU, showing that the enzyme has a length of 559 amino acids and it is a hydrophobic transmembrane protein with its major portion located outside the endoplasmic reticulum membrane. Its secondary structure consists mainly of α-helices and disordered loops, while analysis of the tertiary structure showed that the active region consists of 66 amino acid residues from two polypeptide chains in the homotetrameric structure. Squalene synthase (SQS) is a key enzyme in the MVA pathway that catalyses the condensation of two molecules of famesyl diphosphate (FPP) to form squalene, which then undergoes a series of redox reactions to produce isosteroids and sterols. The levels and activity of SQS determine the yield of the subsequent products of the MVA pathway (Xu et al., 2018b). Wang et al. cloned the complete ORF sequence of FRUSQS, showing a sequence length of 1,230 bp. The SQS protein consists of 409 amino acid residues, with a molecular weight of 46.84 kDa; the protein is a monomeric hydrophilic enzyme localized in the endoplasmic reticulum membrane, with a secondary structure dominated by α-helices, and the active centre situated in a central hydrophobic cavity surrounded by several α-helices, suggesting that the catalytic mechanism may be very similar to that of human SQS. The high expression of the FuSQS gene in FRU bulbs indicates that SQS has an important regulatory role in the FRU alkaloid synthesis pathway, influencing the overall yield of subsequent alkaloid products (Wang et al., 2021). Liao et al. conducted a comparative transcriptomic and metabolomics analysis on four different FRU tissues, namely, the leaves, flowers, stems, and bulbs. They found that imperialine, peimisine and peiminine were the representative bioactive components in FRU bulbs, and a total of 9,217 differentially expressed unigenes were identified among the four different tissues, suggesting that the MVA pathway plays a more important role in the biosynthesis of FRU steroidal alkaloids than the MEP pathway (Liao et al., 2023). Huang et al. using comparative analysis of mevalonate kinase (MK) genes, successfully identified the FRUMK gene, which was found to have an ORF length of 1,158 bp and to encode a 385-residue polypeptide; the strongest similarity was seen with FRC, with a sequence similarity of up to 77.14%. Two key amino acid residues, His198 and Ser137, were also identified, laying a theoretical foundation for the study of the biological role of its gene in the FRU steroidal alkaloid biosynthetic pathway (Huang et al., 2024).

There is an overall lack of genetic information on FRU and few studies have investigated the biosynthesis of steroidal alkaloids involved in the plant. Research on its synthesis-related genes can lay a theoretical foundation for the biosynthesis of steroidal alkaloids and alleviate the current situation of resource scarcity.

### 4.2 Biosynthetic pathway

Qi et al. (2023) proposed that the biosynthetic pathway of isosteroidal alkaloids can be broadly divided into three parts, namely, formation of universal precursors (IPP and DMAPP), cholesterol synthesis, and the formation and secondary modification of isosteroidal alkaloids. Liao et al. (2023) proposed that the biosynthetic pathway of FRU steroidal alkaloids consists of two major stages. In Phase I, the acetyl-CoA produced by glycolysis generates the two units of steroid skeleton formation, IPP and DMAPP, via the MVA pathway, while in Phase II, farnesyl diphosphate synthase (FPS) catalyses the production of FPP.FPP is a precursor of sterols and all non-sterol isoprenoids.

## 5 Pharmacological effects

**FIGURE 7:**
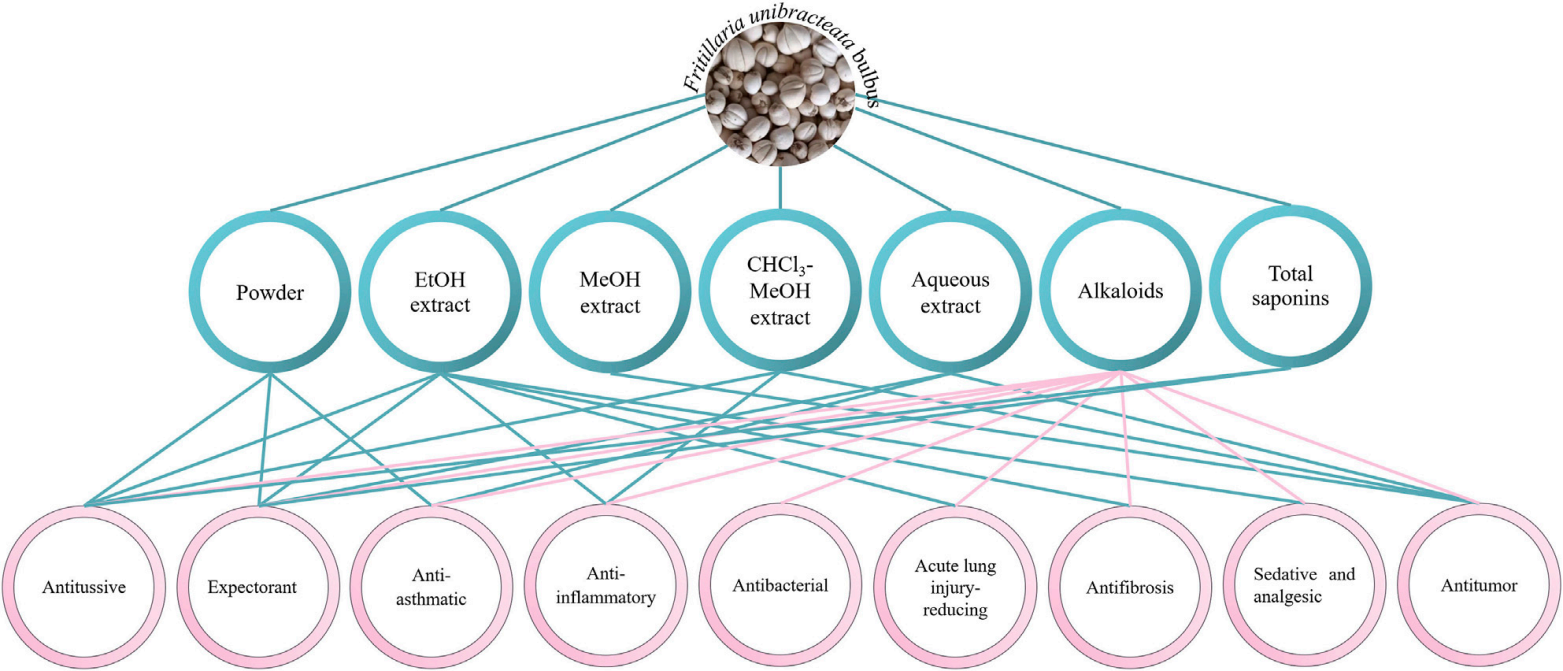
Pharmacological effect diagram of FRU.

*Fritillaria* bulbs have been used as medicine for thousands of years. FRU bulbs have the effects of clearing heat and moistening the lung, resolving phlegm and relieving cough, resolving masses, and eliminating carbuncles (Chinese Pharmacopoeia Commission, 2020). Modern pharmacological studies have shown that FRU has sedative, analgesic, antibacterial, acute lung injury–reducing, antifibrosis, antitumor, antioxidant, and antimalarial effects. The pharmacological effects of FRU are shown in Figure 7.

### 5.1 Antitussive and expectorant effects

Cough is one of the most common symptoms of many diseases and the most common reason for patients visiting respiratory medicine centers (Ke and Ji, 2011). Most herbal remedies have few or no side effects compared with synthetic drugs. FRU contains numerous chemical components that have varying efficacies. Alkaloids have an obvious antitussive effect, which involves inhibiting the cough center without inhibition of the respiratory center. This mechanism is extremely beneficial in the treatment of chronic bronchitis complicated with pneumonia (Xu and Xu, 2013). Studies have also reported the obvious expectorant effect of total saponins.

Several studies have confirmed the antitussive and expectorant effects of FRU. Ding et al. (2010) enrolled patients with acute bronchitis as experimental subjects and compared the differences in cough- and phlegm-resolving efficacies of FRD and FRU. FRU was more efficacious than FRD in clearing heat and moistening the lungs, relieving cough, and resolving phlegm. An *in vivo* study suggested that imperialine, chuanbeinone, isoverticine, peimine, and peiminine from FRU could significantly prolong the cough latency, reduce the cough frequency, promote the production of phenol red in the trachea, and achieve the effect of relieving cough and phlegm in mice (Wang et al., 2011; Wang et al., 2012). Huang et al. (2018) compared the antitussive effects of 5 varieties of CBM alkaloids using a mouse model of ammonia-induced cough and found that FRT (wild and cultivated) and Songbei, Qingbei, and Lubei alkaloids had antitussive effects.

### 5.2 Anti-inflammatory effects

**FIGURE 8:**
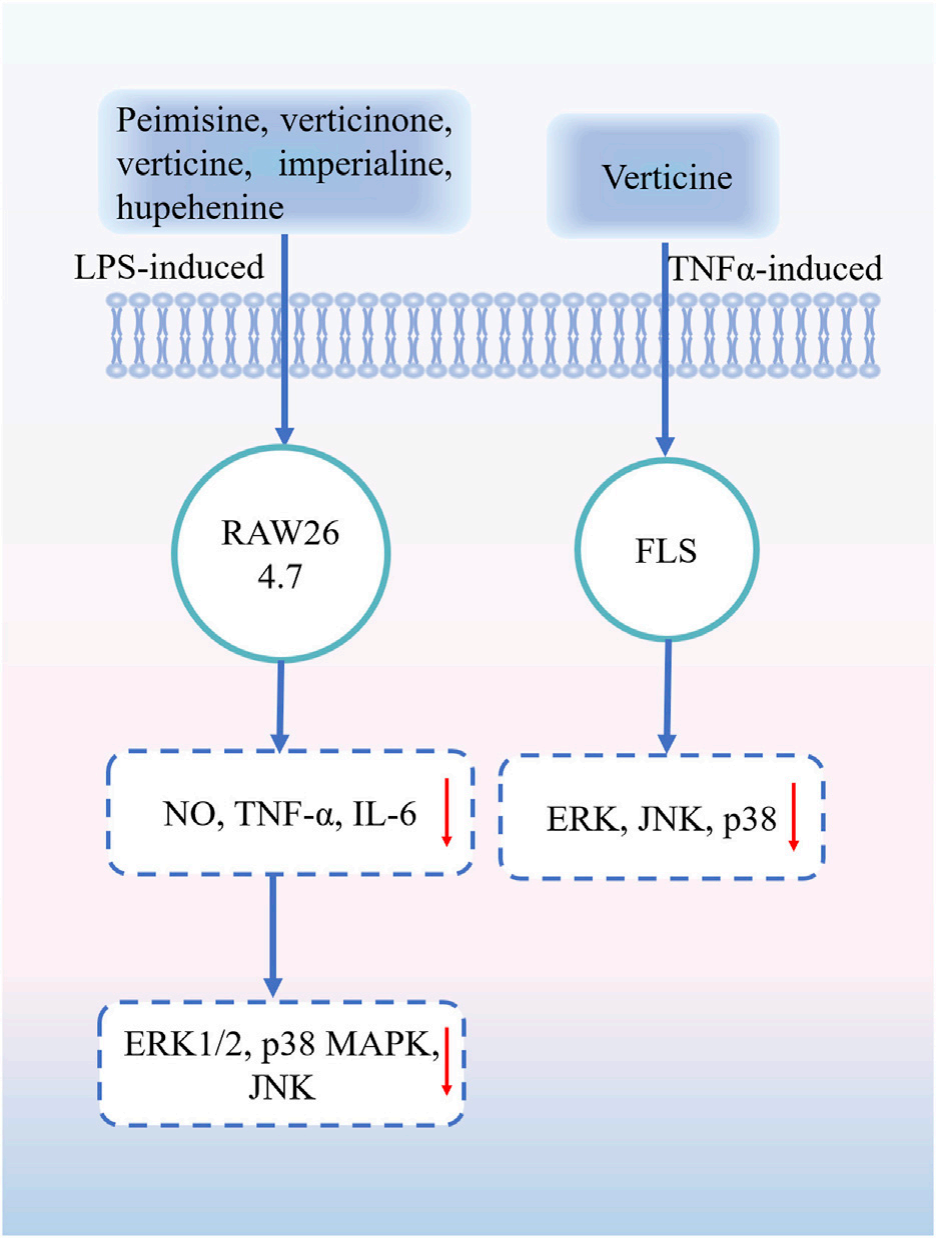
Part of the anti-inflammatory mechanism of FRU. Abbreviations: lipopolysaccharide (LPS); nitric oxide (NO); tumor necrosis factor α (TNF-α); interleukin-6 (IL-6); extracellular signal-regulated kinase (ERK1/2); p38 mitogen-activated protein kinase (p38 MAPK); c-Jun N-terminal kinase (JNK); fibroblast-like synoviocytes (FLSs). (“↓” represents the decrease).

Inflammation is the response of the body to harmful external stimuli and infection. Persistent or chronic inflammation can affect the normal metabolic processes of an organism and induce conditions such as cardiovascular disease, nervous system disease, and inflammation (Zhang et al., 2021) Plants alkaloids are an important class of molecules with anti-inflammatory activity that inhibit the expression of pro-inflammatory factors including cytokines, lipid mediators, histamine, and enzymes involved in the inflammatory response (Peng et al., 2019; Souza et al., 2020; Lu et al., 2022). The anti-inflammatory activity of alkaloids in FRC bulbs was determined by establishing various validated animal models, and the effect of total alkaloids on respiratory inflammation was evaluated using a lipopolysaccharide (LPS)-induced model of acute lung injury. FRC alkaloids exhibited a significant anti-inflammatory activity, providing a systematic pharmacological basis for the anti-inflammatory effect of FRC (Wang and Chen, 2016). Owing to the anti-inflammatory effect, FRC extracts can also prevent ear edema in mice and increase the output of phenol red to the trachea (Xu et al., 2019). An *in vitro* study found that peimisine, verticinone, verticine, imperialine, and hupehenine in the bulbs of FRC can downregulate the levels of inflammatory mediators in LPS-induced RAW264.7 macrophages by inhibiting mitogen-activated protein kinase (MAPK) phosphorylation (Liu et al., 2019a). Peimine can significantly inhibit synovitis and bone destruction in rats with collagen-induced arthritis, and peimine can significantly inhibit tumor necrosis factor (TNF)-α-induced destructive behavior of arthritic fibroblast-like synoviocytes (FLSs). Molecular mechanism studies showed that peimine could significantly inhibit MAPK activation (xtracellular signal-regulated kinase, c-Jun N-terminal kinase, and p38) in TNF-α-induced arthritic FLSs (Zhou et al., 2022). The alkaloidal components of *Fritillaria* have significant anti-inflammatory effects; however, their mechanisms of action remain to be further elucidated so that the medicinal value of *Fritillaria* plants can be more effectively developed. Part of the anti-inflammatory mechanism of FRU is shown in Figure 8.

### 5.3 Anti-asthmatic effects

Asthma is the most common chronic respiratory disease that endangers patients’ lives. Its pathogenesis has not yet been clarified and could be life-threatening in severe cases. *Fritillaria* alkaloids exert antitussive and anti-asthmatic effects by selectively inhibiting tracheal M receptors (Eglen et al., 1992; Atta-ur-Rahman et al., 1998). Li et al. (2017) used a mouse model of asthma to study airway remodeling and found that CBM could effectively inhibit airway inflammation and improve symptoms of asthma in model mice. They attributed the mechanism of action to be related to the reduction of matrix metalloproteinases 2 and 9 and tissue inhibitor of metalloproteinase-1. Peimine, peiminine, imperialine, sipeimine-3-O-β-D-glucoside, and puqietinone had a strong inhibitory effect on the contraction of carbachol-induced isolated guinea pig trachea, leading to antitussive and anti-asthmatic effects (Zhou et al., 2003). An *in vivo* study found that FRC could alleviate inflammation and T-helper (Th)1/Th2 imbalance in mice with ovalbumin-sensitized asthma. The mechanism may be related to the inhibition of janus kinase 3/signal transducer and activator of ranscription6 (JAK3/STAT6) signaling pathway activation (Hou et al., 2022).

### 5.4 Sedative and analgesic effects

Pain is a common reason for individuals to seek medical assistance (Fan et al., 2021). Extracting and isolating active ingredients with analgesic effects from plants is a hot research topic. A study suggests that verticinone may relieve inflammation-related pain and cancer-related neuropathic pain via both peripheral and central mechanisms, and may be partially involved in the sedative effect. These findings indicate the potential of verticinone as a novel sedative and analgesic drug without any dependence liability; however, its exact mechanism of action needs to be further elucidated (Xu et al., 2011). *Fritillaria* alkaloids have good sedative and analgesic effects. Thus, future studies could focus on determining their mechanisms of action, exploring their potential in a clinical setting, and promoting the rational use of this valuable medicinal material.

### 5.5 Antibacterial effects

The total alkaloids of FRU have a bacteriostatic effect on *S. aureus*, making this a subject worthy of further exploration (Chen et al., 2012). It could provide a richer resource material for the development of natural and novel antibiotics. In a study on the screening, identification, and determination of antibacterial activity of endophytic actinomycetes from FRU, 5 strains of *Streptomyces* that could produce active alkaloids were screened. Among them, the CS4 strain could inhibit the growth of *E. coli* and *Pseudomonas aeruginosa*. The CS5 strain could inhibit the growth of *E. coli*, *S. aureus*, and *Candida albicans* (Chen et al., 2017). This is helpful in exploring the potential application value of FRU and providing a reference for the development of novel and natural drugs. There are relatively few studies on the antibacterial effect of FRU; therefore, its antibacterial effect could be further explored in subsequent studies.

### 5.6 Acute lung injury–reducing and antifibrotic effects

**FIGURE 9:**
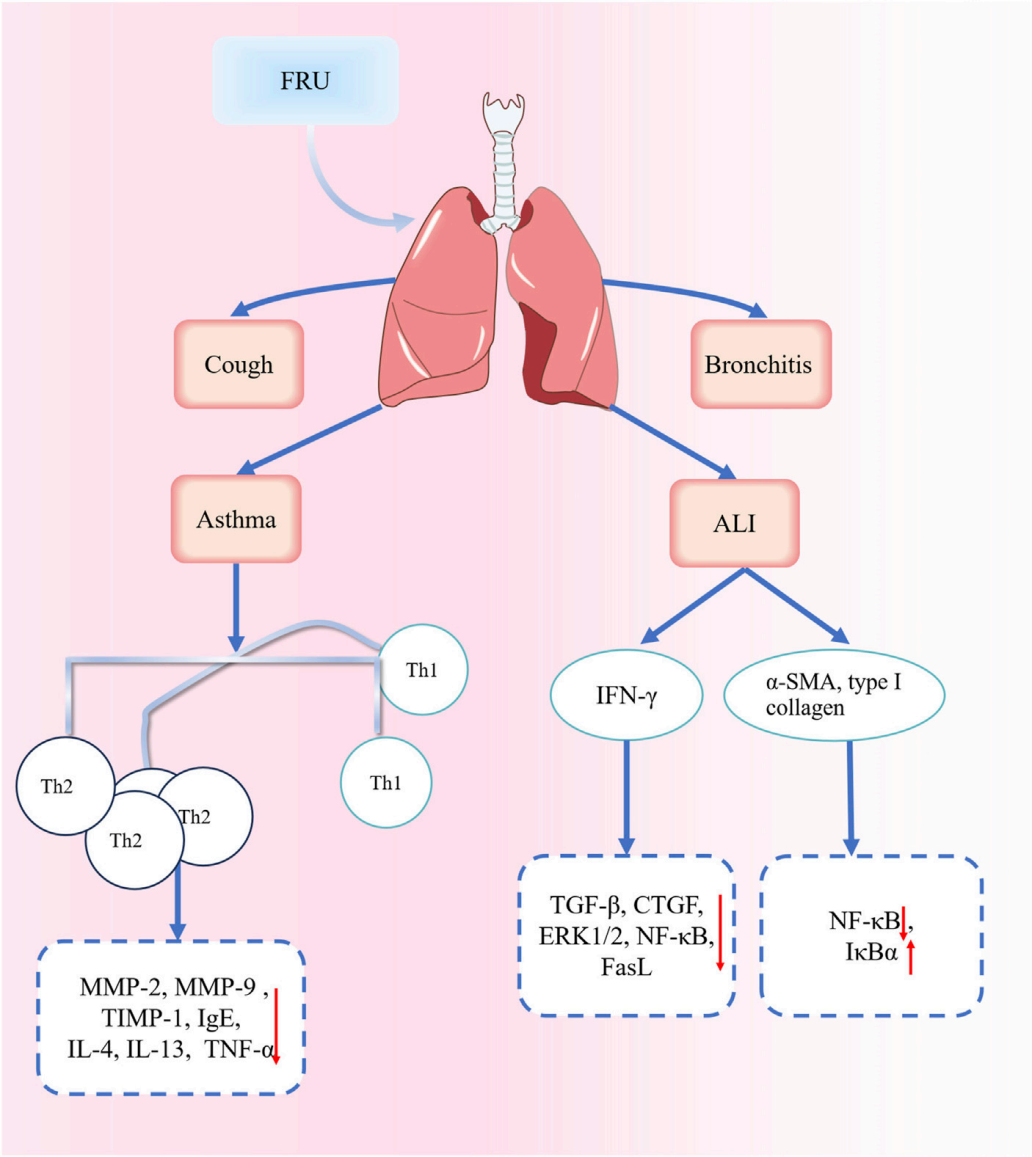
Part of the mechanisms involved in the treatment of respiratory diseases with FRU. Abbreviations: matrix metalloproteinases (MMP); tissue inhibitor of metalloproteinase-1 (TIMP-1); Immunoglobulin E (IgE); Interleukin 4 (IL-4); Interleukin 13 (IL-13); T-helper-1 (Th1); Acute lung injury (ALI); interferon-γ (IFN-γ); transforming growth factor-β (TGF-β); connective tissue growth factor (CTGF); nuclear factor kappa-B (NF-κB); Fas ligand (Fasl); α-smooth muscle actin (α-SMA); Inhibitor kappa B alpha (IκBα). (“↑” represents the increase, “↓” represents the decrease).

Acute lung injury can lead to acute hypoxic respiratory insufficiency or respiratory failure (Bian et al., 2021). Acute respiratory distress syndrome and pulmonary fibrosis can sometimes occur, leading to a sharp decline in lung function. The resulting lung injury is irreversible (Zhan and Shen, 2022). By decreasing circulating interferon-γ levels and inhibiting the signal transduction pathways such as the transforming growth factor-β (TGF-β), connective tissue growth factor (CTGF), extracellular signal-regulated kinase 1/2 (ERK1/2), nuclear factor kappa-B (NF-κB), and FasL pathways, peiminine can alleviate pulmonary inflammation and pulmonary fibrosis in a rat model of bleomycin-induced lung injury (Guo et al., 2013). A study confirmed that the ethanol extract of FRC bulbs could inhibit the expression of α-smooth muscle actin and type I collagen in lung tissues, reduce the pNF-κB/NF-κB ratio, and upregulate the protein expression of Inhibitor kappa B alpha (IκBα). Collectively, these findings suggest that the extract can reduce pulmonary fibrosis in rats by inhibiting NF-κB signaling pathway activation (Yuan et al., 2022). The alkaloidal components of *Fritillaria* show great potential in treating acute lung injury and pulmonary fibrosis. Future studies could focus on the mechanisms of action and provide novel candidate drugs to treat acute lung injury and pulmonary fibrosis. Part of the FRU mechanism for treating respiratory diseases is shown in Figure 9.

### 5.7 Antitumor effects

**FIGURE 10:**
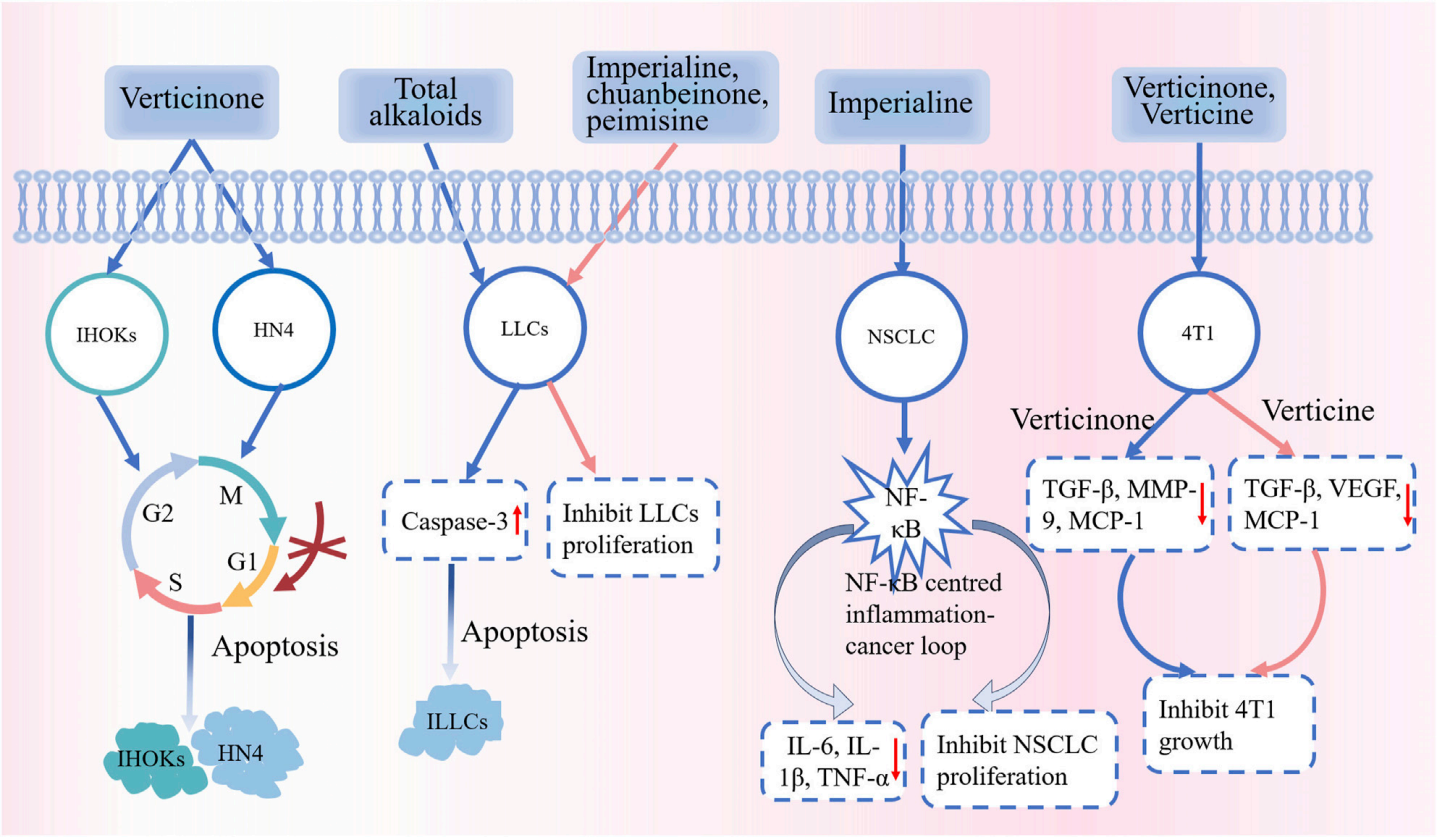
Part of the anti-tumour mechanism of FRU. Abbreviations: immortalized human oral keratinocytes (IHOKs); oral cancer cells (HN4); Lewis lung carcinoma cells (LICs); non-small cell lung cancer (NSCLC); interleukin-1β (IL-1β); monocyte chemotactic protein-1 (MCP-1); vascular endothelial growth factor (VEGF); Mouse Breast Carcinoma 4T1 Cells (4T1). (“↑” represents the increase, “↓” represents the decrease).

The incidence and mortality of cancer are currently on the rise, which poses a major public health problem globally. Existing methods to treat cancers are associated with several side effects and unsatisfactory outcomes (Li et al., 2010). Therefore, research on the antitumor effects of phytochemicals has been gradually attracting attention. *Fritillaria* alkaloids are known to exert antitumor effects. Some studies have shown that verticinone can inhibit the proliferation of oral malignant keratinocyte cells in a dose- and time-dependent manner, and the primary mechanisms of action are inhibition of the G0G1 cell cycle and induction of apoptosis (Yun et al., 2008). The total alkaloids of CBM have potent antitumor activity and low *in vivo* toxicity during the induction of apoptosis in Lewis lung cancer cells. Moreover, the proliferation of Lewis lung cancer cells *in vitro* could be significantly inhibited by imperialine, chuanbeinone, and peimisine (Wang et al., 2014). Lin et al. (2020) studied the anti-nonsmall cell lung cancer (NSCLC) effect of imperialine *in vitro* and *in vivo*. The *in vitro* and *in vivo* anti-NSCLC effects of imperialine have been reported; its mechanism may be related to the NF-κB–centered inflammation–cancer feedback loop. Imperialine has extremely low side effects. Studies have also reported the therapeutic effects of *Fritillaria* alkaloids on breast cancer, ovarian cancer, endometrial cancer, and gastric cancer. The latest data from the World Health Organization indicate breast cancer as the leading variant of cancer worldwide. Zhang et al. (2014) have reported the significant inhibitory effects of peimine and peiminine on 4T1 breast cancer cells. These compounds could effectively decrease the secretion of inflammation-related factors and their relative mRNA expression, thereby exerting an anti-inflammatory effect. FRC can inhibit the growth of ovarian cancer cells and endometrial cancer cells and reduce their invasive potential. FRC acts by reducing the expression of phosphorylated IκBα, thereby decreasing NFκB activation and then inhibiting NFκB-activated metastasis-promoting proteins, eventually inhibiting the growth and invasion of cancer cells (Kavandi et al., 2013). An *in vitro* study showed that peimine could significantly reduce the activity and migration of MKN-45 gastric cancer cells (Zhang et al., 2022). Part of the anti-tumour mechanism of FRU is shown in Figure 10.

### 5.8 Other effects

Modern pharmacological studies indicate that *Fritillaria* alkaloids also exert antioxidant and antimalarial effects (Liu et al., 2019b; Bora et al., 2023).

## 6 Clinical applications

As early as *Shennong’s Classic of Materia Medica* (about 200 A.D.), CBM has been recorded to be used as medicine for more than 2000 years (Liu et al., 2023a). FRU is one of the main original source plants of CBM, and Chinese medicinal preparations involve the use of CBM, which is widely used in the treatment of cough, phlegm, lung fever, pneumonia, asthma, bronchitis, and tumours, among other diseases. In the Chinese Pharmacopoeia 2020 Edition, there are dozens of proprietary Chinese medicines containing Chuanbeimu, including Chuanbei Zhikelu, Chuanbei Pipa Syrup, Chuanbei Xueli Ointment, Chuanbei Pipa Syrup, Chuanbei Pipa Dropping Pills, Xiao’er Zhisou Syrup, Niuhuang Shedan Chuanbei Liquid and Compound Chuanbeijing Tablets.

Bei Mu can be used as single herb or compound for the medicine. For improved efficacy in clinical practice, it is often combined with various traditional Chinese medicines depending on the disease, syndrome, and symptoms, and has been shown to have both good efficacy and safety, with significant potential for clinical application (Fan et al., 2020). Ding et al. used a multicenter, randomized, double-blind, controlled trial to observe differences in the efficacy of FRU and FRD in the treatment of acute bronchitis (phlegm-heat cough). The patients were divided into the FRU and FRD groups, with both groups receiving oral doses of 5 capsules (0.4 g/capsule) three times a day. The results showed that while there was no significant difference between the two groups in terms of efficacy in relieving cough and resolving phlegm, FRU was better than FRD in alleviating the phlegm-heat cough of the patients, especially in clearing heat, as well as moistening the lungs, relieving cough and resolving phlegm. Two of the 104 patients treated with FRD experienced adverse effects, while none of the 106 patients who received FRU showed adverse reactions. However, the differences in the incidence of adverse effects between the two groups were not statistically significant, and there was no incidence of serious adverse events during the test period, indicating good overall safety (Ding et al., 2010). In a randomized controlled clinical study, Li compared the effects of Beimu Gualou Powder and azithromycin in children with pneumonia caused by *mycoplasma* infection. The control group with azithromycin (10 mg/kg, intravenous drip once daily for 4 d), stopping for 3 days and followed by 3 days of azithromycin (5 mg/kg, intravenous drip once daily) while the treatment group received Beimu Gualou Powder. The results showed that the total effective rate in the treatment group of 97.7%, which differed significantly from the total effective rate of 87.2% in the control group, demonstrating the efficacy of Beimu Gualou Powder. There was no significant difference in the incidence of adverse reactions between the groups (9/47 in the control group vs. 10/48 in the treatment group), with all adverse effects returning to normal after treatment, indicating the safety of the formulation (Li, 2012). Qu et al. used a randomized, double-blind, controlled, multicenter trial to observe the efficacy of Zhike Chuanbei Pipa Dropping Pills in the treatment of cough after the common cold. The test group received oral doses of Zhike Chuanbei Pipa Dropping Pills (30 mg/pill, 6 pills/times, 3 times/d) and Compound Guazijin Granules Mimetic (20 g/bag, 1 bag/times, 3 times/d), while the control group was treated with orally administered Compound Guazijin Granules (20 g/bag, 1 bag/times, 3 times/d) and Zhike Chuanbei Pipa Dropping Pills simulant (30 mg/pill, 6 pills/dose, 3 times/d). The results showed that the total effective rate of the experimental group was 92.50%, which was higher than that of the control group (75.00%), and the difference was statistically significant. Thus, the use of Zhike Chuanbei Pipa Dropping Pills for treating cough after the common cold was effective. The incidence of adverse reactions was 2.50% in both the treatment and control groups, with any adverse effects disappearing at the end of treatment, indicating good safety (Qu et al., 2022). The common formulations are shown in Table 2.

**TABLE 2:** Common CBM-based herbal formulations.

Disease type	Therapeutic agents	References
Cough, abundant phlegm	FRC combined with *Ophiopogon japonicus, Scrophularia ningpoensis*	Wu and Zhang. (2009)
	FRC combined with *Amygdalus communis vas*	Fan et al. (2020)
	FRC combined with *Adenophora stricta, Eriobotrya japonica*	Liu and Gao. (1999)
	FRC combined with *Platycodon grandiflorum, Glycyrrhiza uralensis*	Xu et al. (2008)
Pulmonary heat	FRC combined with *Nelumbo nucifera, Lilium brownii*	Su and Yang (2006)
	FRC combined with *Cynanchum stauntonii*	Li et al. (2011)
Infantile pneumonia	FRC combined with *GuaLou, Platycodon grandiflorum*	Li. (2012)
Asthma	FRC combined with of *Amygdalus communis vas, Asiatic plantain* herb	Zhuo (2009)
	FRC combined with *Anemarrhena asphodeloides Bunge*	Chen (2006)
Chronic bronchitis	FRC combined with *Datura metel* L., *Pinellia*	Li et al. (2017b)
	FRC combined with *Ephedra*	Wang and Wang (1993)
	FRC combined with *Platycodon grandiflorum, Eriobotrya japonica*	Zhou (2014)
Breast cancer, lung cancer	FRC combined with *GuaLou*	Wang et al. (2016a); Yang and Bian (2014)

## 7 Breeding and cultivation technology

Due to the extremely high medicinal value of FRU, the market demand has been increasing, leading to over-exploitation and the endangerment of wild resources. Moreover, its specific habitat, long seed dormancy period, and low natural germination rate mean that wild resources are far from being able to meet the market demand. The plant is now classified under the national third-level endangered protection of medicinal herbs. Thus, to promote the reasonable use of the FRU resources, many researchers have investigated techniques for the breeding and cultivation technology of FRU.

### 7.1 Tissue culture propagation technology

The tissue culture of Bei Mu generally uses bulbs as explants. Li et al. investigated tissue culture techniques associated with FRU bulb regeneration. The results showed that FRU bulb slices could induce calluses in MS, B5 and H media, of which the rate of tissue healing in MS medium was higher, while high concentrations of NAA inhibited the development of small bulbs. The use of the 1.5 NAA+0.05 Kt combination was found to be more effective for the induction of small bulbs (Li et al., 1995). Cai et al. (1992) studied culture methods for FRU and showed that the growth rate was highest in shaken liquid culture, less in solid culture, and lowest in rotary bed liquid culture. The alkaloid content was found to be highest in shaken liquid culture (0.06753%), second highest in solid culture (0.06685%), and lowest in rotary bed liquid culture (0.05025%). Li et al. (2003) induced embryoids of tissue culture fritillaria at low temperatures. It was found that the embryoids treated at low temperature for 40 days produced the highest number of seedlings. However, although the tissue culture of FRU has been the subject of extensive research, it still cannot be applied to industrial production.

### 7.2 Cultivation technology

Xu et al. (2018a) analysed changes in the fresh and dry weights, as well as the moisture, ash, and total alkaloid contents of cultivated FRC and FRU samples from different growth years, and concluded that the best harvesting period for both was in the middle and late July. Fang et al. (2023) found that altitude was the main factor affecting the ecological factors of FRU under different conditions of origin and cultivation by monitoring the dynamic changes of the ecological factors of FRU, indicating that genuine medicinal materials must be cultivated and wild nurtured in genuine producing areas. Annual precipitation is the second most important ecological factor after altitude that affects the distribution of FRU, with an optimal precipitation of 400–1,400 mm. Too much or too little rainfall can reduce the emergence of seedling as well as cause death during later growth stages, thus, rain-sheltered cultivation in greenhouses provides precise control of the water supply (Peng et al., 2021). Peng et al. (2024) compared and analysed the effects of six different light-quality conditions on the growth and development, stress resistance, and active ingredient contents of isolated FRU seedlings, determining the biomass, physiological parameters, and medicinal ingredient concentrations of FRU, and found that treatment with blue light was beneficial, allowing FRU to maintain stable vegetative growth and increase the total alkaloid content, laying the foundation for large-scale production of FRU. Liu et al. (2023b) used metagenomics and metabolomics techniques to monitor changes in the inter-root microbial communities and metabolites of FRU over 1, 2, and 3 years of cultivation, and found that the microbial compositions differed significantly among FRU groups from the 3 year from 2020 to 2022. These changes were associated with trends of reduced relative abundance of beneficial microorganisms, increased relative abundance trends in the of harmful microorganisms, and significant changes in the metabolite compositions of the soil at the inter-root level. The relative abundance of some beneficial metabolites was observed to decrease overall. Deng et al. (2022) conducted a field fertilization trial of 5-year-old FRU based on a secondary universal rotary combination design using herb yield and total ash, alcohol-soluble leachate, and total alkaloid content as indices, developing a fertilizer regimen for high quality and high yield of FRU with N ranging from 163.82 to 190.77 kg/hm^2^, P_2_O_5_ ranging from 541.75 to 720.25 kg/hm^2^, and K_2_O ranging from 337.49 to 454.52 kg/hm^2^. FRU requires specific soil conditions for effective cultivation, requiring looser soil with a high humus content, while clay or sandy loam is not suitable (Peng et al., 2021). Wang et al. (2006) applied the Geographic Information System for the Analysis of the Suitability of Chinese Materia Medica Origins to analyse suitable areas for FRU cultivation using temperature, altitude, rainfall, and soil type as indicators, leading to the identification of suitable regions for FRU cultivation across the country. Liu et al. used the latitude and longitude of the geographical distribution points of FRU, combined with 26 ecological factors, and used the Maximum Entropy Model (MaxEnt) in conjunction with a Geographic Information System to predict the potential distribution areas of FRU in China. The results showed that these areas were mainly located in western and northern Sichuan, southern Qinghai, and southern Gansu, and that there were five main ecological factors contributing to the FRU distribution, namely, the altitude (40.8%), the average annual precipitation (28%), the maximum temperature in January (7.1%), the average temperature in the driest season (6.6%), and the average daily diurnal temperature difference (6.6%), with the altitude of 2,700–4,500 m, annual average annual precipitation (28%), and the daily average diurnal temperature difference (6.6%) all located in the same region as the distribution area. Thus, regions with an altitude of 2,700–4,500 m and mean annual precipitation of 400–1,400 mm are the most suitable ecological niches for growing FRU (Liu et al., 2018). Zhao et al. (2023) used the MaxEnt model to predict potentially suitable habitats using factors such as bioclimatic variables, elevation, soil factors, and human activities, finding that human activities had a greater impact (3.61%) on the suitability of an area for FRU growth, and that the suitability of areas that were otherwise considered highly, moderately, or poorly suitable habitats was reduced by 21.63, 48.91, and 30.84%, respectively, by the presence of human activities. Currently, the breeding and cultivation of FRU is still in the stage of small-scale research and there is as yet no large-scale cultivation. Altitude, light, temperature, humidity, fertilisation, and soil are the main factors affecting the cultivation of FRU, so further research on the cultivation technology of FRU is needed to alleviate the current situation of resource shortage.

## 8 Conclusion and future prospects

We have reviewed the botanical characterization, resource distribution, phytochemistry, biosynthesis, pharmacological effects, clinical application, and breeding techniques of FRU, thereby providing a basis for the development, utilization, and resource protection of FRU. In addition to its antitussive, expectorant, and anti-asthmatic effects, modern pharmacological studies have shown that FRU also has antioxidant, antibacterial, sedative, analgesic, acute lung injury–alleviating, antimalarial, antitumor, and other effects as well as high medicinal value. However, as a mainstream commodity of FRC, FRU is now endangered in terms of resources and is far from meeting the market demand. Therefore, the following aspects need attention:

First, FRU cultivation is currently at the stage of small-scale research. Further in-depth research is needed on breeding techniques and the protection of resources, as well as on the quality of the cultivated products in terms of traits, chemical composition, and therapeutic efficacy, to alleviate the endangered status of FRU and to ensure sustainable utilization of current FRU resources. Second, the chemical composition of traditional Chinese medicines is diverse and their effects result from the synergism of various active ingredients. Most studies on FRU currently focus on alkaloids; however, as its alkaloid content is extremely low, and it is thus necessary to investigate other components such as saponins and assess synergistic effects among the different components, in order to promote the development and utilization of FRU. Third, FRU exerts numerous pharmacological effects, thereby showing potential for clinical application. These effects have to be further evaluated, as mechanistic studies on the active compounds of FRU are limited. For example, previous studies have shown that the alkaloids of FRU have obvious anti-inflammatory effects; however, the specific targets of action and the inflammatory signaling pathway need to be further studied to promote its clinical use and new drug development. Lastly, in recent years, due to the increase in market demand, the price of FRU has increased to US$690/kg. To derive more profit, merchants often adulterate FRU with other varieties of cheaper Bei Mu herbs, which are often similar in appearance and difficult to distinguish morphologically. The presence of Bei Mu powder is extremely difficult morphologically, and there is thus a need to explore convenient, fast, more accurate and sensitive identification methods. Existing analytical methods for the determination of doping include chromatographic methods such as TLC (Zhai et al., 2017), HPLC (Wang et al., 2016), and LC-MS (Yang et al., 2021), spectroscopic methods such as NIR (Xie et al., 2022), laser-induced breakdown spectroscopy (LIBS) (Wei et al., 2023) and terahertz spectroscopy (Xu et al., 2021), and electronic sensing techniques (Feng et al., 2020). The chromatographic method, however, is based on the chemical composition, and can easily be affected by factors such as the planting and storage environments, and the method is cumbersome to operate. Spectroscopic methods tend to be complicated and time-consuming in terms of sample pretreatment, and the spectral signal is easily disturbed. The use of electronic sensing has received increasing attention in recent years, including the electronic eye (E-eye), electronic nose (E-nose) and electronic tongue (E-tongue) (Zhou et al., 2017; Feng et al., 2020). E-eye simulates the human eye’s perception of samples to differentiate different samples by color, the E-nose simulates the human olfactory organ to identify samples by odor, and the E-tongue simulates the use of the human tongue to identify samples (Zhou et al., 2017). Electronic sensing is associated with simple sample pre-processing and high methodological sensitivity, but the technology still requires the development of highly sensitive sensor materials and the exploration of easier methods for data analysis. Bioinformatics is now beginning to be used in the identification of traditional Chinese medicines, and DNA barcoding and biochip techniques could be used in the future in the assessment of FRU adulteration. In future research, it is necessary to establish a method to identify the adulteration of FRU from multiple aspects of traits, chemical composition, and biology to enhance quality control and improve the stability and safety of the quality of the herbs.
